# Optimizing ultraviolet B radiation exposure to prevent vitamin D deficiency among pregnant women in the tropical zone: report from cohort study on vitamin D status and its impact during pregnancy in Indonesia

**DOI:** 10.1186/s12884-019-2306-7

**Published:** 2019-06-21

**Authors:** Raden Tina Dewi Judistiani, Sefita Aryuti Nirmala, Meilia Rahmawati, Reni Ghrahani, Yessika Adelwin Natalia, Adhi Kristianto Sugianli, Agnes Rengga Indrati, Oki Suwarsa, Budi Setiabudiawan

**Affiliations:** 1 Public Health Department, Faculty of Medicine Universitas Padjadjaran, Jalan Eijkman 38, Bandung, Jawa Barat 40161 Indonesia; 2 Centre of Immunology Studies, Faculty of Medicine Universitas Padjadjaran, Bandung, Indonesia; 3 Master in Midwifery Program, Faculty of Medicine Universitas Padjadjaran, Bandung, Indonesia; 4 Department of Child Health, Faculty of Medicine Universitas Padjadjaran, Bandung, Indonesia; 5 dr Hasan Sadikin Hospital, Bandung, Indonesia; 6 Clinical Pathology Department, Faculty of Medicine Universitas Padjadjaran, Bandung, Indonesia; 7 Department of Dermatovenereology, Faculty of Medicine Universitas Padjadjaran, Bandung, Indonesia

## Abstract

**Background:**

Vitamin D deficiency during pregnancy carries potential threat to fetal well being. Natural conversion of vitamin D in the skin can be facilitated by direct ultra violet B (UVB) radiation, but the effect is reduced by wearing umbrellas, clothes, or sunblock cream. Muslim women wear hijab that allows only face and hands to be seen. With increasing proportion of muslim women wearing hijab and the lack of vitamin D fortification and fish consumption in Indonesia, it poses a problem for vitamin D deficiency among pregnant women. This study aimed at finding the best timing of UVB exposure and the duration of exposure which can be suggested to prevent vitamin D deficiency among pregnant women, for those wearing hijab or not.

**Methods:**

This study recruited 304 pregnant women in the first trimester, 75–76 women from 4 cities of the most populated province, West Java, Indonesia which represented 70–80% percent of pregnancy per year. A 3-day notes on duration, time and type of outdoor activity and the clothing wore by the women were collected. UVB intensity radiation were obtained. Calculation on body surface area exposed to direct UVB radiation and UVB radiation intensity were done. Measurement of vitamin D level in sera were done on the same week.

**Results:**

The median of maternal sera vitamin D level was 13.6 ng/mL and the mean exposed area was around 0.48 m2 or 18.59% of total body surface area. Radiation intensity reached its peak around 10.00 and 13.00, but the mean duration of exposure to UVB during this window was lower than expected. Significant correlation was found between maternal sera vitamin D level and exposed body surface area (r = 0.36, *p* < 0.002) or percentage of exposed body surface (r = 0.39, *p* < 0.001) and radiation intensity (r = 0.15, *p* = 0.029). Further analysis showed that duration of exposure to UVB should be longer for pregnant women wearing hijab as compared to women without hijab.

**Conclusion:**

This study suggested that the best timing to get UVB exposure was between 10.00–13.00, with longer duration for women wearing hijab (64.5 vs 37.5 min) of continuous exposure per day.

**Electronic supplementary material:**

The online version of this article (10.1186/s12884-019-2306-7) contains supplementary material, which is available to authorized users.

## Background

Vitamin D deficiency has been recognized as a global public health problem and it plays a wide role in health and disease prevention [1]. Previously, it has been presumed that vitamin D deficiency will be more common in temperate climate region such as North America and Europe [2]. However, the occurrence of vitamin D deficiency is also common in countries around the equator line or tropical zone such as South Asia and Southeast Asia [3, 4]. Some countries have vitamin D deficiency prevalence of more than 40% among adult population. The prevalence was even higher in pregnant women, which affected more than 60% of them [1]. It may be due to imbalance of supply and demand during pregnancy.

Vitamin D, a lipophilic hormone, presents in two forms: natural ergocalciferol (vitamin D2), mainly derived from plant sources through radiation of ergosterol produced by yeasts, and cholecalciferol (vitamin D3), mainly produced in the skin through conversion by ultraviolet B (UVB) radiation. Other sources come from animal products such as fatty fish, mushrooms, egg yolks, liver, and dairy products [5, 6]. UVB radiation is an important factor to convert 7-dehydrocholesterol in the skin into pre-vitamin D, isomerized by body heat into vitamin D3 (cholecalciferol) then transported by the blood to the liver, where it is *converted* to 25-hydroxyvitamin *D* (25-OH Vit *D*) [7].

Increased calcium and vitamin D requirements during pregnancy increased the risk of vitamin D deficiency in pregnant women [8]. It has been reported that low maternal vitamin D increased the risk of adverse pregnancy outcomes such as preeclampsia, gestational diabetes mellitus, preterm birth, and small for gestational age babies [9–12]. The use of supplementations or food fortification have been recommended. However, a systematic review reported that concurrent use of vitamin D and calcium supplementation increased the risk of preterm birth [13]. Achieving optimal level of vitamin D through adequate exposure to sunlight is then considered safer.

Optimal sunlight exposure in different region could be influenced by many causes: environmental factors such as solar zenith angle, clouds, ozone, surface reflection, altitude; and human factors such as age, skin pigmentation, duration of exposure, use of sunscreen, type of clothing, total body surface area exposed to sun, and body mass index [7, 14]. Moreover, in many Asian and Middle East countries, cultural and religion practice highly influence daily exposure to sunlight [15, 16]. Sun-seeking behaviour is also uncommon in tropical Asian populations due to warm climate most of the year and cultural view that fair skin is associated with beauty [3]. Unfortunately, reports regarding vitamin D status in Indonesian population are scarce. A study conducted by Setiati et al. in 2008 reported the prevalence of vitamin D deficiency among Indonesian elderly women aged 60 years and older in nursing care was around 35.1%. Most deficiency cases occurred in patients that went out-door only once a week, wore veil, and exposed to sun around 30–60 min a week [17]. However, there has not been any study which investigated the association between UVB radiation exposure and vitamin D level among pregnant women in Indonesia. The aim of this study was to explore the effects of exposure to UVB in daily activity, maternal clothing style on vitamin D level in the first trimester.

## Methods

This study was set to begin the Cohort Study on Vitamin D Status and Its Impact During Pregnancy in Indonesia, conducted from July 2016. West Java Province was chosen as it has the largest population of pregnant women, it is located from 5^o^50’ – 7^o^50’ S to 104^o^48’ – 108^o^48’ E [18] Women recuited form Bandung, Sukabumi, Waled and Cimahi to represent different geographical areas of the province. The midwives offered the pregnant women to participate in this study on their first encounter, explained the whole procedure and complete information. Pregnant women were met at their clinic, health centres, or at Posyandu, a voluntary-cadre lead post which is conducted once a month. Interested candidates were referred to the appointed hospital for ultrasound examination by the attending obstetricians. Pregnant women were recruited if they were (1) resident of the city, (2) in gestational age between 10 and 14 weeks as confirmed by ultrasonography and (3) had a normal singleton pregnancy.

Every eligible women gave her consent to participate in the study and allow publication of this study results.

The number of sample needed for this a study was 97, based on the formula for correlation study as follow

$$ \mathrm{n}={\left\{\frac{Z_{\alpha +{Z}_{\beta }}}{0,5 In\left[\left(1+r\right)/\left(1-r\right)\right]}\right\}}^2+3 $$

n = sample number, Zα = 1.64, Zβ= 1.28, r=0.3

We suspected that vitamin D deficiency was high in Indonesia. In order to increase the chances of getting more pregnant women with normal level of vitamin D and also to reduce bias, we recruited subjects more than 3 times the number of samples needed, 75 from each city.

Interviews to obtain demographic data, obstetric history were conducted by trained midwives during recruitment. The participating women were trained to record their daily activity which had direct exposure to sunlight. The data consisted of date, duration and hour of the day activity, and the clothing they used during their activity in the following 3 days. The record were checked and recollected by the midwives.

Calculation of total body surface area (TBSA) in meter square was done using Mosteller formula [19] as shown below.

$$ \mathrm{TBSA}\ \left({\mathrm{m}}^2\right)=\sqrt{\frac{\mathrm{height}\ \left(\mathrm{cm}\right)\ast \mathrm{weight}\ \left(\mathrm{kg}\right)}{3600}} $$

We also calculated the Percentage of body area which were exposed to sunlight using combined Mosteller and Wallace formula [20]. An example of calculation based on the type of clothing and the practice of wearing hijab is shown in Table 1.

**Table 1:** Calculations of total body surface area exposed to ultra violet B radiation: Combination of Mosteller and Wallace Formula

	Clothing	Exposed area	Percentage
Without hijab	Long sleeved top, long bottom (trousers or skirt)	Head, neck, hands, feet	9 + (2*2.5) + (2*2) = 18
	Short sleeved top, short bottom (trousers or skirt)	Head, neck, part of upper arms, lower arms, hands, part of upper thighs, knees, calves, shinbones, feet	9 + (2*6) + (2*13.5) = 48
	Short sleeved top, long bottom (trousers or skirt)	Head, neck, part of upper arms, lower arms, hands, feet	9 + (2*6) + (2*2) = 25
With hijab	Long top and bottom without socks	Face, hands, feet	4.5 + (2*2.5) + (2*2) = 13,5
	Long top and bottom with socks	Face, hands	3 + (2*2.5) = 8

To obtain data for individual vitamin D level, 10 cc of blood from median cubiti vein was drown. Approximately 5 cc was used for this study and the serum was separated. The remaining blood was used for complete blood count and hepatitis screening as in routine antenatal screening. The sera for vitamin D analysis were transferred in cool box to Dr. Hasan Sadikin Hospital in Bandung. The whole sera were stored in -20^o^ Celcius to maintain its quality, awaiting for collection of at least 80 samples which might take more than a month or two. After thawing, vitamin D level measurement were done using enzyme-linked immunosorbent assay (ELISA). The kit for ELISA was VIDAS® 25 OH Vitamin D Total from bioMérieux SA. The minimum reading for detection was 8.1 ng/mL. Any lower results were rounded as 8 ng/mL.

In the interpretation of the result, the women were further classified based on their vitamin D status into: deficient (< 20 ng/mL), insufficient (21–29 ng/mL), and normal (≥30 ng/mL) as described by American Endocrinology Society [21].

Hourly UV radiation intensity data was obtained by a machine, Vantage Pro 2®, from the Indonesian National Institute of Aeronautics and Space office which was located in Bandung. According the standard from Indonesian Government only one machine was available for every province. Records were obtained from September 2016 until January 2017 that matched the period of study participants’ recruitment. To establish the dosage of UV radiation, the intensity in watt/m^2^ was converted into minimal erythema dose (MED) per hour. One MED is defined as the amount of UVB radiation that will produce minimal erythema (redness caused engorgement of capillaries) of an individual’s skin within a few hours following exposure [22].

The main analysis was performed using IBM SPSS Statistics for Windows version 24 (IBM Corp., Armonk, N.Y., USA). Descriptive statistics were presented as frequencies and proportions for categorical variables, median and interquartile range for continuous variables. Spearman’s rank correlation was used to assess the association among total body surface area exposed to sun, UV intensity, and maternal sera vitamin D level. Further we used multinomial logistic regression analyses to analyze the associations among factors influencing UVB exposure and serum vitamin D categories. Normal vitamin D level was considered as the base category to which the other two categories were compared. Age, parity, pre-pregnancy BMI, education, and the use of sunscreen were included a priori in the adjusted model as potential confounders.

## Results

**Fig. 1:**
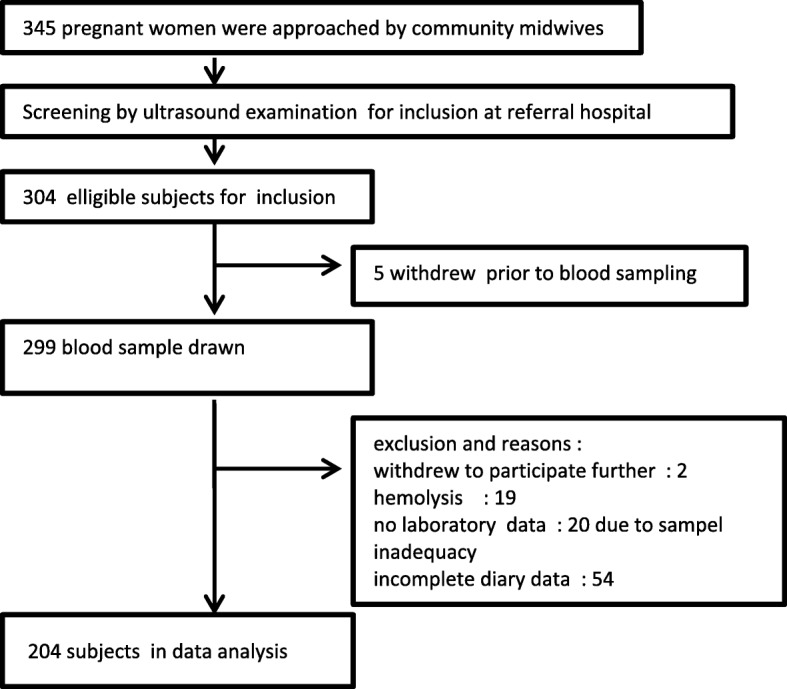
Selection of study participants

The community midwives were able to approach 345 pregnant women at the community level. These pregnant women were referred to hospital for further screening. Three hundred and four women fulfilled our inclusion criteria by ultrasound, but then there were 5 women who withdrew their participation prior to blood sampling. There were some more reasons for exclusions after that as shown in Fig. 1.

The final sample of this study was 204 subjects. Seventy four women (36.37%) did not wear hijab. The mean age of these women was 28.4 years old and body mass index (BMI) 23.7 kg/m^2^. The mean for total duration of outdoor activity of all these women was 69.75 min between 06.00–18.00 h each day. The duration of each activity lasted between 5 to 10 min, and a sub total of 29.1 min were done between 10.00–13.00. The type of outdoor activity in all groups were similar, which were walking to grocery stores, drying clothes under the sun, watering garden or dropping and picking up children from school. No sport activity was recorded.

Their characteristics were shown in Table 2.

**Table 2:** Characteristics of Pregnant Women

Characteristics	Vitamin D status			Total	Mean (SD)	*p*-value
	Deficient	Insufficient	Normal			
	(*n* = 164)	(*n* = 33)	(*n* = 7)			
Use of sunscreen						0.19
No	145	26	7	178		
Yes	19	7	0	26		
Age					28.4 (5.83)	0.27
< 20 years	11	1	0	12		
20–34 years	130	27	4	161		
≥ 35 years	23	5	3	31		
Education						< 0.001
Elementary school	38	14	7	59		
Middle school	45	12	0	57		
High school	54	5	0	59		
Diploma/bachelor	27	2	0	29		
Occupation						0.18
Housewife	113	29	6	148		
Civil servant	37	2	1	40		
Others	14	2	0	16		
Pre-pregnancy body mass index (kg/m^2^)^a^					23.7 (4.84)	0.41
Underweight (< 18.5)	14	2	1	17		
Normal (18.5–24.99)	83	32	4	119		
Overweight (25–29.99)	32	5	2	39		
Obese (> 30)	25	4	0	29		
Parity						0.17
0	70	10	0	80		
1	54	13	4	71		
≥ 2	40	10	3	53		
Use of veil (*hijab*)						0.002
No	77	25	6	108		
Yes	87	8	1	96		

*P*-value was calculated using Chi-square test.

^a^Body mass index clasification by WHO 2018, available from http://apps.who.int/bmi/index.jsp?introPage=intro_3.h

Due to the limitation of ELISA tool, every value less than 8.1 ng/mL was recorded as 8 ng/mL. Maternal vitamin D was ranged from 8.0 to 39.0 ng/mL. The mean (SD) was 14.7 (6.5) ng/mL, the median (interquartile range/IQR) of 13.6 (10) ng/mL. We found that 42 women (20.5%) had very low vitamin D level (< 8.1 ng/mL).

Based on daily maternal clothing data, we calculated the TBSA exposed to sunlight using Mosteller formula and found the median TBSA in our study participants around 0.48 m^2^ (IQR = 0.46). With simplified Wallace formula, we found that the median percentage of exposed body area was 18.59% (IQR = 19.5).

Data on daily ultraviolet radiation from the sun during the months of observations were obtained, it is presented in Table 3 It showed that in general, the optimum radiation intensity increased gradually from 10.00 to reach its peak at 13.00. It became a bit lower between 13.00–15.000 and reduced gradually further after 15.00. The lowest intensity was recorded between 06.00–07.00 and between 17.00–18.00. Table 3 also showed that January 2017 had the lowest point of UVB radiation. After conversion of radiation intensity from watt/m^2^ to MED per hour unit, we found that the median of UVB intensity was around 0.39 MED/hour (IQR = 0.43).

**Table 3:** Average hourly UVB intensity in September 2016 – March 2017

Time (hour)	Average hourly UVB intensity (watt/m^2^)						
	September	October	November	December	January	February	March
00.00 – 06.00	0	0	0	0	0	0	0
06.00 – 07.00	0.2	0.9	1.2	0.7	0.01	0.47	3.37
07.00 – 08.00	4.9	4	2.7	3.7	0.18	2.99	6.35
08.00 – 09.00	5.7	5.7	4.1	5.2	0.3	3.85	7.79
09.00 – 10.00	7.8	8.2	6.5	7.5	0.47	6.95	10.4
10.00 – 11.00	9.9	11	9	9.7	0.63	9.65	12.6
11.00 – 12.00	9.7	12	8.6	12	0.67	11.9	13.5
12.00 – 13.00	9.7	10	8.9	13	0.63	12.7	13.3
13.00 – 14.00	8	9.4	8	12	0.54	11	11.2
14.00 – 15.00	7.4	7.1	6.1	9	0.45	8.16	9.75
15.00 – 16.00	5.9	5.9	3.9	6.6	0.3	5.39	7.34
16.00 – 17.00	5.6	3.3	3.4	4.1	0.16	3.01	5.35
17.00 – 18.00	1.3	1	1.2	3.5	0.06	1.02	1.42
18.00 – 00.00	0	0	0	0	0	0	0

Spearman’s rank correlation showed that there was significant correlation between maternal serum vitamin D level with TBSA, percentage of body area exposed to sun, and UVB intensity as shown in Table 4.

**Table 4:** Correlation of total body surface area, percentage of body area exposed, and ultra violet B intensity, with maternal serum vitamin D level

	Correlation coefficient (r_s_)	*P*-value
TBSA (m^2^) exposed to sun with maternal vitamin D	0.360	< 0.001
Percentage of body area exposed to sun with maternal vitamin D	0.390	< 0.001
UVB intensity (MED/hour) with maternal vitamin D	0.153	0.029

Univariable multinomial logistic regression detected decreased odds of vitamin D deficiency with increased TBSA (OR (95% CI) = 1.89E-107 (2.24E-204 - 1.6E-10), *p*-value = 0.03) and percentage of body area (OR (95% CI) = 0.93 (0.87–0.99), *p*-value = 0.02) exposed to sun. However, the significant association diminished after adjustment with potential confounders as shown in Table 5.

**Table 5:** Associations among factors influencing UV radiation and maternal serum vitamin D level. Data collection form (Additional file 1)

Exposure	Outcome	cOR (95% CI)	p-value	aOR (95% CI)	p-value
TBSA (m^2^) exposed to sun	normal	ref			
	deficiency	1.89E-107 (2.24E-204 - 1.6E-10)	0.03	3.76E-93 (1.39E-226 - 1.02E+41)	0.18
	insufficiency	3.22E-22 (3.35E-124 - 3.12E+80)	0.68	2.14-16 (3.97E-152 - 1.15E+120)	0.82
Percentage of body area exposed to sun	normal	ref			
	deficiency	0.93 (0.87-0.99)	0.02	0.94 (0.86-1.02)	0.12
	insufficiency	0.99 (0.93-1.05)	0.69	0.99 (0.91-1.08)	0.83
UVB intensity (MED/hour)	normal	ref			
	deficiency	0.74 (0.23-2.4)	0.23	1.85 (0.26-13.13)	0.54
	insufficiency	1.23 (0.37-4.13)	0.37	2.98 (0.41-21.49)	0.28

*cOR* crude odds ratio

*aOR* adjusted odds ratio by age, parity, pre-pregnancy BMI, education, and the use of sunscreen

## Discussion

This study demonstrated that majority (80.4%) of pregnant women in of West Java had vitamin D deficiency. Only educational level was found significantly different in baseline characteristics between women with vitamin D deficiency compared to women with insufficiency or normal vitamin D level.

More than 70% of pregnant women in this study did not use sunblock. Although sunblock use have been promoted to prevent cutaneous carcinogenesis, a recent study in Belgium reported that a 50+ sun protection factors (SPF) sunblock significantly decreased cutaneous vitamin D production following single UVB exposure independent of the TBSA with minimal effect to circulating 25-OH Vit D [23]. Regulation of serum vitamin D level is a complex process and many factors influence the bioavailability of circulating vitamin *D* [2, 24]. High proportion of vitamin D deficiency despite rare use of sunblock found in our study suggested that other endogenous or exogenous processes influenced vitamin D level.

Most pregnant women in this study were at the optimal reproductive age, i.e. between 20 and 34 years old. No significant difference of vitamin D level was found among age groups, most likely due to relatively younger and short range of age (16–43 years). The effect of age on vitamin D level will be more prominent in elderly people since they have thinner dermal layer, and consequently less ability of synthesizing vitamin D [25].

Overweight or obese individuals are prone to vitamin D deficiency. Vitamin D is a fat soluble vitamin and fat deposition all over the body would disturb transportation and conversion of previtamin D_3_ into provitamin D_3._ Thus, overweight or obese individuals have reduced capacity of vitamin D synthesis [26, 27]. Since more than 50% of our study participants had normal pre-pregnancy BMI, no significant difference was observed.

This study found that there was a significant difference of education level between vitamin D deficiency group and insufficient-normal group. Lower educational level has been associated with vitamin D deficiency in Saudi Arabia and Poland [28, 29]. Similar observation was found in this study since more than half of the participants (116 women, 56.86%) had education level of middle school or lower. This would influence dietary pattern and other daily life style related to individual vitamin D status [30–32]. The free supplement for pregnant women from the government did not provide supplement with vitamin D. Very few subjects (9 women) stated that they consumed vitamin D containing supplement for less than a month, but they were still deficient in vitamin D. Previous reports from our cohort had shown that the proportion of anemia increased by trimester among women with colecalciferol deficiency and that colecalciferol level in blood was associated with better fetal growth as indicated by biparietal diameter and abominal circumference [18, 33]. It is very likely that the changes in life style, which is exposing adequate skin to sun at appropriate time and duration to enhance vitamin D conversion may improve fetal growth.

The median of maternal serum vitamin D in this report was lower than the previous report (15.34 vs 13.6 ng/mL), far below the normal level of 20 ng/mL [18]. The commercially available vitamin D supplements in Indonesia were expensive, it can be 10 times higher than the original price at the exporting countries, which makes it less affordable for most pregnant women.

Significant associations of vitamin D level with biparietal diameter and abdominal circumference were consistent after adjustment with maternal age, pre-pregnancy body mass index, parity, serum ferritin level, and hemoglobin level [33].

This would make the result of this study very important for the Indonesian government, to change recommendation and improve the health promotion program. As Indonesia is a country in tropical region, sunlight should be available all year long with abundant ultra violet B, but caution should be put as this study also showed there was a lowest point for ultraviolet B radiation in January 2017. Adequate UVB for vitamin D conversion also depends on the duration of exposure and TBSA exposed. Based on the UVB intensity data, it was best to be exposed to the sun from 10.00 until 13.00. However, most subjects tend to do less outdoor activities during that period, which was also reported in Pakistan and Italy [34, 35]. In countries with large Muslim population, the religious practice of wearing hijab is common and it has been demonstrated to be an independent factor for vitamin D deficiency in the Middle East and South Asia region [36–39]. Similar finding was also found in our study as previously shown in Table 1.

UVB radiation data retrieved from the local institute of aeronautics and space was recorded in watt/m^2^. However, human skin sensitivity to sun exposure varies depending on skin pigmentation [40]. Based on Fitzpatrick classification, majority (80–90%) of Indonesian population is classified as type III and IV with melano-competent features [41]. Previous study at Hasan Sadikin Hospital in Bandung, Indonesia, reported that for skin type III and IV the UVB radiation intensity to achieve 1 MED was around 69.7 J/cm^2^, which was similar to findings in India, despite the most common skin type reported in India was skin type V, which was darker than type III and IV [42].

Hollick et al. reported that sun exposure to face, arms, and hands could achieve adequate dosage of UVB radiation [43]. When it was converted into percentage of body area exposed to sun based on combined Mosteller and Wallace formula (Table 1), the minimum area that needs UVB radiation was around 22.5%. More than half of pregnant women in this study did not achieve adequate UVB radiation since the median of body area exposed to sun was only 18.59%.

This study found that the median of UVB intensity was 0.39 MED/hour. In order to achieve 1 MED, daily exposure to sun requires approximately 2.5 h., but according to Hollick, the minimum duration for obtaining adequate vitamin D conversion was only 25% of time duration to achieve 1 MED [43]. Thus, the minimum duration of sun exposure was around 37.5 min per day. This amount of exposure would not be valid for women using hijab since the body area exposed to sun was only 13.5% or less. Therefore, women with hijab should increase their exposure time to at least 64.5 min per day.

This study found significant correlations between the width or percentage of body surface area exposed to sun, UVB intensity, and maternal sera vitamin D level.

Multinomial regression analysis failed to support the associations, which may indicate that larger samples are needed to identify the most influencing factor for vitamin D level among these pregnant women.

Several other limitations could have influenced the results of this study. Firstly, this study could not detect vitamin D level below 8.1 ng/mL due to limitation of the ELISA methods. Second, UVB intensity data was only available from Bandung, as the office would place the tool only in every capital of the Indonesian provinces. In this study it was used as an approximation for other cities in West Java. On the other hand, the strength of this study lies on its population-based design and multiple locations that represented several geographical areas, north to south, west to east and urban/rural area, of West Java. We were able to produce information on the urgency of promoting outdoor activity between the optimum hours of utilizing the energy from the sun to prevent vitamin D deficiency among pregnant women, however a randomized clinical trial is still needed to assess its efectivity.

## Conclusion

This study found that vitamin D deficiency was prevalent among pregnant women in West Java, Indonesia. There were significant correlations between maternal vitamin D level with TBSA, percentage of body area exposed to sun, and UVB intensity. The best time of achieving UVB exposure between 10.00 and 13.00 on a daily basis, and that outdoor activity within this time period should be encouraged. Pregnant women without hijab can be advised to have continuous exposure for approximately 37.5 min per day, while for women with hijab the advisable duration was around 64.5 min per day. A carefully designed clinical trial may be proposed to prove that these findings could be incorporated in maternity education and health promotion to prevent vitamin D deficiency.

## Additional file

Additional file 1: Data collection form. (DOCX 14 kb)

## Abbreviations

BMI: Body mass index
ELISA: Enzyme-linked immunosorbent assay
IQR: Interquartile range
MED: Minimal erythemal dose
SPF: Sun protecting factor
TBSA: Total body surface area
UVB: Ultraviolet B
