# Supplementary material for: Optimizing ultraviolet B radiation exposure to prevent vitamin D deficiency among pregnant women in the tropical zone: report from cohort study on vitamin D status and its impact during pregnancy in Indonesia
Source: BMC Pregnancy Childbirth. 2019 Jun 21;19:209. doi: 10.1186/s12884-019-2306-7 (PMC6588851; doi:10.1186/s12884-019-2306-7)
Supplement: Supplementary file 1 — Data collection form. (DOCX 14 kb) [file 12884_2019_2306_MOESM1_ESM.docx]

Data collection Form

Subject code :

Name : …………………………………………….. .. Date of birth : ………………………………

Address : ………………………………………………………………………………………………………………….

City : …………………………………………………. ID number : ……………………………….

Phone : ………………………………………………….

Recruited by : ………………………………………….

Education : ……………………………………………. Occupation : …………………………………

Pre pregnancy Weight : ……… kg , Height : …….. cm

Obstetric history : Grav …… Par ….. Ab ……..

First Day of Last Mestrual Period : ……………………

First date of ultrasound : ...... (dd)/ ……. (mo)/………..(yy) gestational age : …… weeks ….. day

Fetal Biometry and Laboratory result

| Date of examination | CRL | BPD | HC | AC | Hb | Vitamin D | Ferritin |
| --- | --- | --- | --- | --- | --- | --- | --- |
|  |  |  |  |  |  |  |  |
|  |  |  |  |  |  |  |  |
|  |  |  |  |  |  |  |  |
|  |  |  |  |  |  |  |  |
|  |  |  |  |  |  |  |  |
|  |  |  |  |  |  |  |  |
|  |  |  |  |  |  |  |  |
|  |  |  |  |  |  |  |  |

Obstetric complication (if any) : ..........

Daily exposure to sunshine (please write down the time and duration of exposure, as well as the outfit/clothing or umbrella that you wear. Indicate when you put on sun block or sun protection cream)

Date ……........................ Hour...…… to …...……. Duration : …… minutes

Outfit : ………….............................................................................................................................

Sun block / sun protection : ......................................................................................................

What kind of activity that you do : ...........................................................................................
